# Effect of dietary supplementation of spray-dried plasma on performance and semen quality in aging broiler breeders

**DOI:** 10.1590/1984-3143-AR2024-0111

**Published:** 2025-06-30

**Authors:** Carlos Alexandre Granghelli, Mário Henrique Scapin Lopes, Luís Rangel, Joy Campbell, Javier Polo, Joe Crenshaw, Eneiva Carla Carvalho Celeghini, Pedro Nacib Jorge-Neto, Helena Lage Ferreira, Vinicius Santos Moura, Jennifer Soutter Motta, Cristiane Soares da Silva Araujo, Lucio Francelino Araujo

**Affiliations:** 1 Departamento de Zootecnia, Faculdade de Zootecnia e Engenharia de Alimentos – FZEA, Universidade de São Paulo – USP, Pirassununga, SP, Brasil; 2 APC, LLC, Ankeny, IA, USA; 3 Departamento de Reprodução Animal, Faculdade de Medicina Veterinária e Zootecnia – FMVZ, Universidade de São Paulo – USP, Pirassununga, SP, Brasil; 4 Departamento de Medicina Veterinária, Faculdade de Zootecnia e Engenharia de Alimentos – FZEA, Universidade de São Paulo – USP, Pirassununga, SP, Brasil; 5 Departamento de Nutrição e Produção Animal, Faculdade de Medicina Veterinária e Zootecnia – FMVZ, Universidade de São Paulo – USP, Pirassununga, SP, Brasil

**Keywords:** breeder age, chick quality, embryonic mortality, sperm cell kinetics, sperm morphology

## Abstract

A total of 216 Cobb 500 broiler breeder hens were randomly distributed across two treatments consisting of diets supplemented with 0 or 1% spray-dried plasma (SDP), resulting in 27 replications per treatment and four birds per pen. In addition, 36 roosters, housed in individual pens, were allocated to the same treatments, with each bird considered a replicate, in order to assess the influence of SDP on semen and hatching characteristics. The experimental diets were fed from 26 weeks until the conclusion of the study, at 65 weeks of age. Semen quality, embryonic mortality and quality of newly hatched chicks were evaluated at 29, 45, and 63 weeks. Hens were inseminated with fresh semen, and eggs were incubated following standard procedures. Semen from 63-week-old roosters had higher sperm concentration than other age groups, while 29-week-old rooster semen exhibited higher progressive motility than semen from older roosters (*P* < 0.001). The 45-week-old roosters had the lowest values for the analyzed semen quality parameters (average path velocity, straight-line velocity and curvilinear velocity). Additionally, sperm from 45-week-old roosters, regardless of SDP supplementation, had fewer total morphological defects than 63-week-old roosters. However, 1% SDP in the diet reduced total sperm cell defects at 63 weeks of age, as well as total sperm head and tail defects (*P* < 0.05) compared to unsupplemented birds. Dietary SDP reduced late embryonic death in 63-week-old breeders (*P* < 0.05). The results demonstrate that 1% SDP in breeder diets reduced late embryonic mortality and sperm cell defects, mainly in aged birds, enhancing the reproductive performance of broiler breeders.

## Introduction

Broiler health and performance are intrinsically related to parent nutrition, age, and reproductive performance (Chang et al., 2016), since the embryo relies on nutrients transferred from the hen to the egg in order to further develop and hatch (Givisiez et al., 2020). Therefore, breeder nutrition must meet the dietary requirements for optimal reproductive performance while also fulfilling the nutrient needs of embryos and newborn chicks (Kakhki et al., 2020; Yang et al., 2021; Nowaczewski et al., 2022). Consequently, alterations in broiler breeder body reserves or dietary composition can affect nutrient deposition and egg quality (Kidd et al., 2013).

In recent decades, broiler production has undergone constant revisions in handling, nutrition and reproductive techniques, which have resulted in improved performance parameters in poultry. However, few studies have been performed to evaluate the effect of breeder hen nutrition on performance of the hens and the impact on their progeny. From a practical point of view, we still have little information on ways to improve broiler breeder reproductive performance through nutrition (Araujo et al., 2018). Hence, investigating ingredients and feed additives that improve breeder intestinal health or enhance offspring quality and hatchability is necessary to advance broiler chicken production further.

Considering the various feed additives available in the poultry industry, spray-dried plasma (SDP) is a potential candidate for use in broiler breeder diets. SDP has been routinely added to pig feed since the 1980s, especially during the post-weaning phase (Coffey and Cromwell, 2001), due to its effect on increasing feed consumption and decreasing diarrhea in weaned pigs (Lawrence et al., 2004), thus reducing the use of antibiotics (Torrallardona et al., 2002; Walters et al., 2019). In broilers affected by an outbreak of necrotic enteritis, an increase in final body weight at market, a decrease in overall feed conversion, and a decrease in mortality for broilers fed diets with SDP have been observed (Bregendahl et al., 2005; Campbell et al., 2006; Walters et al., 2019; Belote et al., 2021). More specifically about reproduction effects, some studies regarding SDP supplementation for transport stressed pregnant mice fed diets with 1, 2, 4 or 8% SDP maintained higher pregnancy rates compared to control mice. Only 1 day after feeding transport stressed pregnant mice diets with 1 or 8% SDP, there was a rapid restoration of Th-1/Th-2 balance in uterine tissue compared to control mice, which had elevated pro-inflammatory cytokines for a longer duration in early pregnancy (Song et al., 2015), thus leading to a reduced oxidative status due to lower inflammatory activity. In late pregnancy, 8% dietary SDP attenuated inflammation in uterine and placenta tissue and reduced lethargic effects induced by injected lipopolysaccharide (Liu et al., 2018).

Although not fully understood, SDP is a protein-rich substance composed of albumin, immunoglobulins, transferrin, growth factors, bioactive peptides, essential amino acids, lipids, enzymes, and other components (Coffey and Cromwell, 2001; van Dijk et al., 2001; Campbell et al., 2008) that improve animal performance and health by maintaining the gastrointestinal tract integrity (Torrallardona et al., 2002; Beski et al., 2015) and modulating the immune system effectiveness (Campbell et al., 2008, 2019). Factors that interfere with rooster fertility and semen quality, such as aging and stress (Fouad et al., 2020), could be mitigated by the rich and diverse nutritional profile of SDP.

It is generally accepted that the hatching process, likewise mammalian pregnancy, causes oxidative stress, and an improvement in the antioxidant defenses of an embryo has the potential to increase hatchability (Rajashree et al., 2014).Under stressful conditions, when free radical production exceeds the protective ability of the antioxidant systems, poultry experience oxidative stress, a condition that causes detrimental consequences on their health (immunosuppression, higher inflammation response), on their reproduction (decreased fertility and hatchability in breeders), and their growth and feed efficiency (Surai, 2018; Bottje, 2019; Pappas et al., 2019).

In this context, little information is available for the use of SDP in broiler breeder diets, despite its dietary use being a potential candidate to improve the offspring productive performance, if supplemented to both breeder hen and roosters, since the beneficial nutrients of SDP could be available in the egg yolk after fertilization. Therefore, the objective of the present study was to assess the effect of dietary inclusion of SDP for broiler breeder hens and roosters on semen quality, fertility rate, embryonic mortality, and hatchling quality, with increasing breeder age.

## Materials and methods

### Birds, diets, and management

The study was conducted at the Poultry Science Laboratory of the School of Veterinary Medicine and Animal Science of the University of Sao Paulo (Pirassununga, SP, Brazil; 21°57’34.3”S 47°27’14.6”W). The experimental protocol was approved by the Ethics Committee on Animal Use of the University of Sao Paulo (protocol no. 7681081220).

A total of 216 *Cobb* 500 slow feathering broiler breeder hens (Initial mean BW: 3178.6 ±368 g) were randomly distributed across two treatment groups in a completely randomized design, resulting in 27 pens per treatment containing four birds per pen. Additionally, a total of 36 *Cobb* 500 roosters (Initial mean BW: 3895.8 ±252 g), kept in individual bird pens within the same environment as the hens, were randomly distributed across the two treatment groups, providing 18 replications per treatment. All rearing procedures followed lineage manuals. Birds were fed corn and soybean meal-based diets supplemented with or without 1% SDP, starting at 26 weeks of age, as further described by Granghelli et al (Granghelli et al., 2023). Breeder hen diets were formulated to contain 2,800 kcal ME/kg with 15% CP from first egg up to 38 weeks of age and 14.5% CP from 38 weeks until the end of the trial. Rooster diets were formulated to contain 2,700 kcal ME/kg with 13% CP.

Each pen contained a through feeder, two nipple drinkers, a nesting box (in hen pens), and wood shavings as bedding. All birds were monitored twice a day, and mortalities and culled birds were recorded, weighed, and removed as they occurred throughout the trial.

### Evaluation of semen quality

Semen quality was evaluated throughout the rooster’s life cycle at 29 (Three weeks after birds started receiving the experimental diets), 45, and 63 weeks of age, and was collected from each rooster by abdominal massage three days before each of the three inseminations. Semen was collected in a conical tube, taking care to ensure that no excreta or blood contaminated the sample. All materials used in semen collection and evaluation were maintained at 30 ºC to avoid thermal shock and alterations in seminal characteristics. The semen ejaculate volume (mL) was recorded. The environment temperature recorded on the day of semen collection was 23.9 ºC at 29 weeks, 10.7 ºC at 45 weeks, and 23.2 ºC at 63 weeks. For evaluation of sperm kinematics parameters, the semen was diluted in a microtube with modified Dulbecco's phosphate-buffered saline (DPBS), aiming for a diluted concentration between 25 and 50x10^6^ sperm/mL. If the concentration exceeded or fell below this range, the dilution process was repeated. The microtube was then closed and gently shaken. Using a 2-200 µL pipette tip, a 3 µL sample was rapidly loaded into a Leja slide chamber (Leja, IMV Technologies – L’Aigle, France) and any external droplets were dried. Eight images were acquired from each sample using a computer-assisted sperm analysis system (CASA), model IVOS II (version MK5; Hamilton Thorne, USA), utilizing the Animal Breeders II software (version 1.13.7; Hamilton Thorne, USA) with specific settings for rooster sperm (Supplementary Material). Cell detection was verified using the “Live Configuration” tool of the software (Jorge-Neto et al., 2024). The kinematic parameters evaluated were: Total motility (TM,%), progressive motility (PM,%), average path velocity (VAP, μm/s), straight-line velocity (VSL, μm/s), curvilinear velocity (VCL, μm/s), wobble from real path (WOB, %), amplitude of lateral head displacement (ALH, μm), beat cross frequency (BCF, Hz), straightness (STR, %) and linearity (LIN,%).

A second semen sample was diluted and fixed in a buffered saline formaldehyde solution (4% formaldehyde in DPBS) for sperm morphology evaluation. The analysis was conducted only for semen from 45- and 63-week-old roosters, using the humid chamber technique, with a droplet (4 μL) of diluted semen placed between the slide and coverslip, observed by differential interference contrast microscopy (DIC, Nikon 80i) under 1,000× magnification, evaluating 200 sperm cells per sample. Abnormal sperm were classified as defects in acrosome, head, middle piece, tail, and total defects, adapted from the methodology proposed by Celeghini et al. (2001), considering the percentage of defects.

### Evaluation of chick quality

Broiler breeder hens were inseminated with fresh semen using a 0.5 mL dose to ensure a minimum concentration of 100 × 10^6^ spermatozoa/mL. Semen was collected by performing an abdominal massage on roosters, with SDP-supplemented hens being inseminated only with a semen pool from SDP-supplemented roosters, and unsupplemented hens with a semen pool from unsupplemented roosters. Both semen quality, semen abnormal cell defects and insemination were perfomed in the same week within the 29, 45 or 63 weeks of age. Eggs were collected up to eight days after insemination for three hatchlings at 29, 45, and 63 weeks. Eggs (n=800) were stored at 18 ºC and then incubated at 37.5 ºC and 55% humidity. Eggs that were soiled, cracked, or deformed were not placed in the incubator. Unhatched eggs were broken to determine the infertile rate. Embryonic mortality was classified by early death (1-7 days), middle death (8-14 days), late death (15-21 days), and pipped eggs with dead embryos.

Chick quality was based on a visual evaluation of a random sample (n=50 females and 50 males) determined by sexing the chicks at hatch. Chicks were inspected for dehydration, red hocks, open navel (large navel, small navel), and unhealed navel (wicks). The body weight (g) of the chicks was measured, as well as length (cm), by placing the chick in lateral decubitus and measuring from the tip of the beak to the end of the middle toe.

### Statistical analysis

For statistical analysis, a 3 × 2 factorial arrangement between age (29, 45, and 63 weeks) and SDP inclusion (0 and 1%) was considered as main effects. The mean value of each variable obtained at 29, 45, and 63 weeks of age was considered. Data were analyzed using JMP Pro v. 14.0 (SAS institute, 2014). For all evaluated parameters, the experimental model included the hen or rooster dietary treatment. All model factors were considered nominal variables. If ANOVA was significant, LSMEANS were separated using a t-test with the significance level set at 0.05. If the interaction effect was significant, main factor effects were not discussed. Chick quality data were analyzed using the Glimmix procedure.

## Results

Supplementing rooster feed with SDP had no effect (*P* > 0.05) on semen volume, concentration, and kinematic characteristics (Table 1). Rooster age affected sperm concentration and quality, since semen collected from roosters at 63 weeks of age had higher sperm concentration than semen collected from previous weeks (*P* < 0.05). Semen from 29-week-old roosters exhibited higher VAP, VSL, VCL, BCF, STR, and LIN compared to other age groups (*P* < 0.001), while semen from 45-week-old roosters had the lowest mean values for all sperm quality characteristics, except for TM (*P* < 0.05).

**Table 1:** Influence of feeding diets with SDP to roosters on semen quality.

**Diet/age**	**TM 1**	**PM**	**VAP (μm/s)**	**VSL (μm/s)**	**VCL (μm/s)**	**WOB (%)**	**ALH (μm)**	**BCF (Hz)**	**STR (%)**	**LIN (%)**	**Sperm concentration (x10^9^ mL^-1^)**	**Semen Volume (mL)**
	**(%)**	**(%)**										
Control	81.25	42.50	60.77	50.45	103.03	57.47	4.88	28.81	78.50	46.56	2.85	0.41
SDP	83.27	39.92	63.59	52.01	107.10	57.62	5.00	29.41	77.22	45.95	3.12	0.43
SEM2	1.73	2.64	1.76	1.92	1.75	0.76	0.06	0.29	0.83	1.06	0.16	0.04
Age (weeks)												
29	82.35	50.35^a^	72.21^a^	62.05^a^	116.45^a^	60.14^a^	5.02^a^	31.60^a^	81.41^a^	50.56^a^	2.39^b^	0.36
45	81.12	33.85^b^	50.76^c^	39.69^c^	90.89^c^	55.39^b^	4.61^b^	27.94^b^	75.08^b^	42.64^b^	2.56^b^	0.52
63	83.31	39.45^ab^	63.56^b^	51.95^b^	107.84^b^	57.10^ab^	5.22^a^	27.79^b^	77.10^b^	45.58^b^	4.02^a^	0.37
SEM	2.07	3.15	2.10	2.39	2.09	0.90	0.08	0.35	0.99	1.27	0.19	0.05
		
Breeder diet	0.414	0.490	0.260	0.568	0.104	0.896	0.168	0.160	0.280	0.687	0.243	0.74
Breeder Age	0.77	0.001	<.0001	<.0001	<.0001	0.0014	<.0001	<.0001	<.0001	0.0001	<.0001	0.06
Breeder diet × age	0.352	0.365	0.146	0.272	0.175	0.157	0.585	0.933	0.598	0.324	0.224	0.078

^1^TM: Total motility; PM: Progressive motility; VAP: Average path velocity; VSL: Straight-line velocity; VCL: curvilinear velocity; WOB: Wobble from real path; ALH: Amplitude of lateral head displacement; BCF: Beat cross frequency; STR: Straightness; LIN: linearity. ^2^SEM: Standard error of the mean. a-c: Means within a column with different superscripts are different by the Tukey test (P < 0.05).

**Figure 1:**
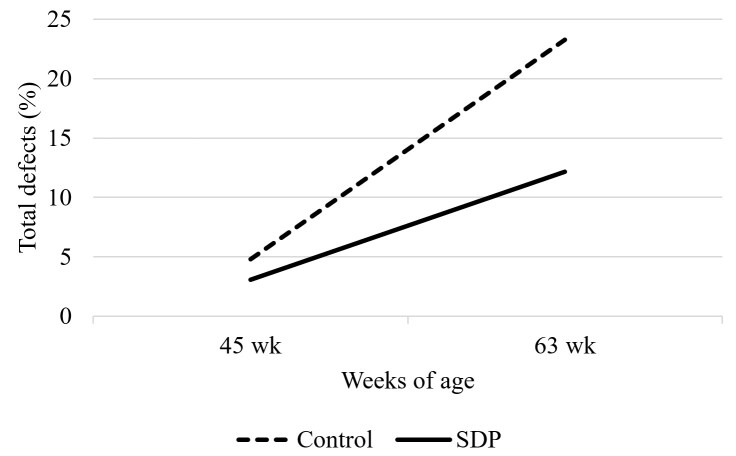
Total defect percentage of abnormal sperm from 45 and 63-week old roosters fed with 0 or 1% SDP.

**Figure 2:**
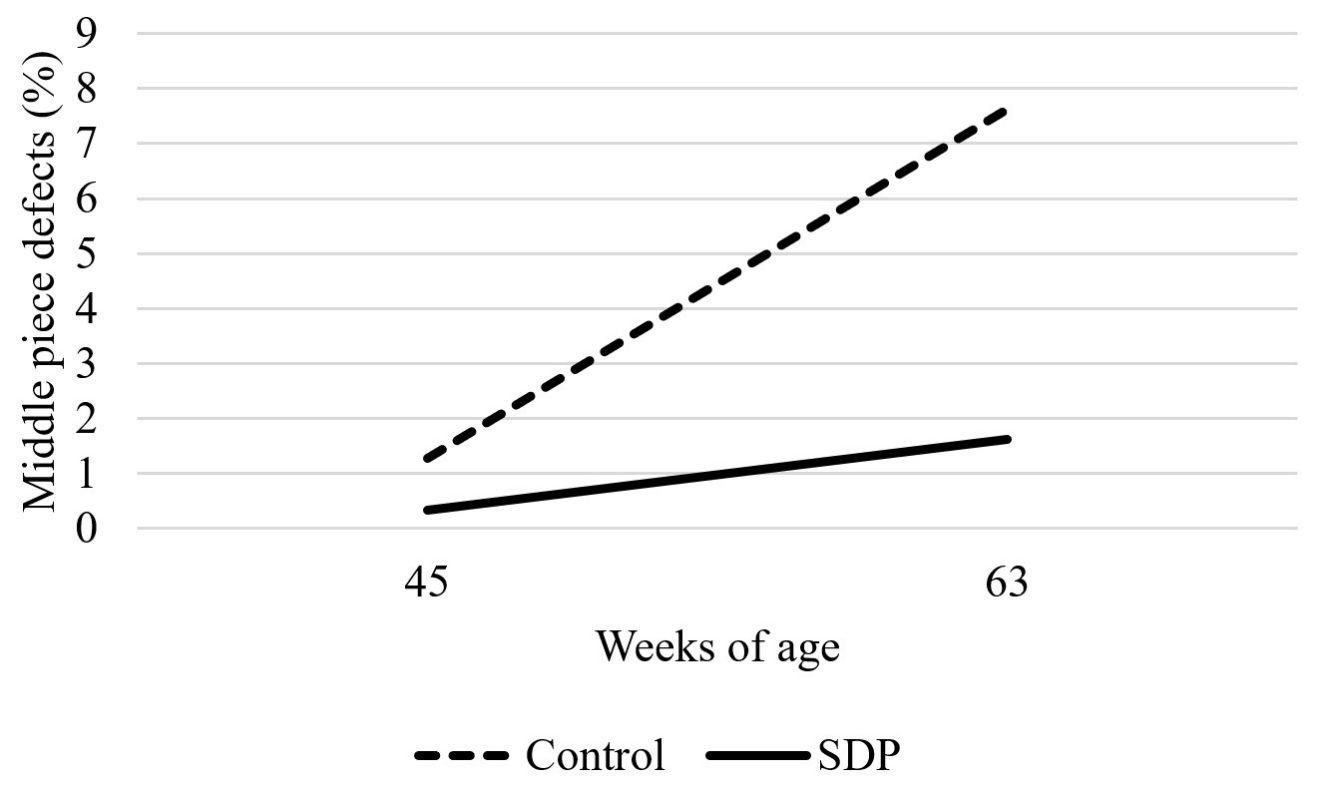
Middle piece defect percentage of abnormal sperm from 45 and 63-week old roosters fed with 0 or 1% SDP.

Regarding sperm morphological alterations, an interaction between factors was observed (Table 2). Sperm from 45-week-old roosters, regardless of SDP supplementation, showed fewer total defects than 63-week-old roosters. SDP supplementation reduced total sperm cell defects at 63 weeks of age compared to the control group of the same age (*P* < 0.05, Figure 1). An interaction regarding defects in the middle piece of the sperm cell was also observed, showing that semen from 63-week-old roosters in the control group had the highest number of middle piece defects among treatments (*P* < 0.05, Figure 2). No effects regarding acrosome defects were observed (*P* > 0.05). Concerning sperm head defects, roosters from the SDP supplemented group had fewer defects (*P* < 0.05) compared to the control group, and 45-week-old roosters had fewer defects than 63-week-old roosters (*P* < 0.001). As for tail defects, semen from roosters supplemented with SDP had fewer defects than the control group (*P* < 0.05).

**Table 2:** Influence of feeding diets with SDP to roosters on spermatic alterations.

**Diet/age**		**Total defects**	**Acrosome defects**	**Head defects**	**Middle piece defects**	**Tail defects**
		------------------------------(%)-----------------------------------				
Control		14.03^a^	0.03	8.31^a^	4.45^a^	1.23^a^
SDP		7.61^b^	0.01	6.21^b^	0.97^b^	0.41^b^
SEM1		1.19	0.01	0.65	0.62	0.27
Age (weeks)						
45		3.94^b^	0.04	2.36^b^	0.77^b^	0.71
63		17.71^a^	6.94	12.14^a^	4.53^a^	0.94
SEM		1.19	0.01	0.64	0.62	0.27
						
Control	45	4.81^c^	0.06	2.62	1.28^b^	0.84
	63	23.26^a^	1.73	14.00	7.63^a^	1.63
SDP	45	3.08^c^	0.02	2.13	0.33^b^	0.58
	63	12.15^b^	5.20	10.28	1.62^b^	0.25
SEM		1.68	0.02	0.90	0.88	0.38
						
				Probability		
Breeder diet		<.0001	0.50	0.02	<.0001	0.03
Breeder Age		<.0001	0.08	<.0001	<.0001	0.56
Breeder diet × age		0.007	0.50	0.07	0.006	0.15

^1^ SEM: Standard error of the mean. a-c: Means within a column with different superscripts are different by the Tukey test (P < 0.05).

**Figure 3:**
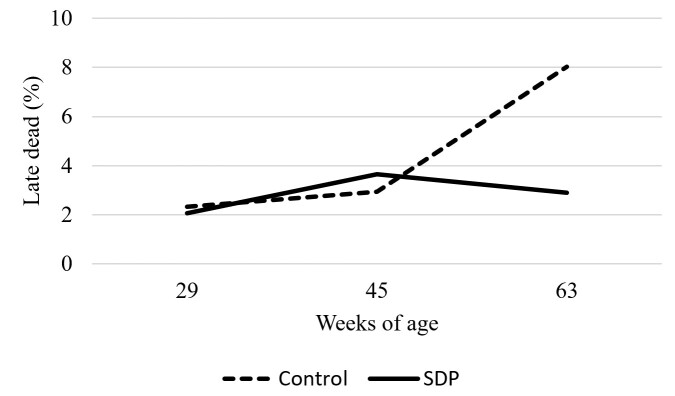
Late embryonic mortality in eggs laid by 29, 45 and 63-week old hens fed with 0 or 1% SDP.

Infertility rates, early dead, mid dead, and pipped egg percentages were not affected by treatments (*P* > 0.05; Table 3). However, an interaction between age groups and SDP supplementation was observed during the late dead analysis, showing that dietary inclusion of SDP reduced the percentage of late embryonic deaths compared to the control group at 63 weeks of age (*P* < 0.05, Figure 3).

**Table 3:** Influence of feeding diets with SDP to roosters and breeders on embryonic mortality.

**Diet/age**		**Infertile**	**Early dead (1-7d)**	**Mid dead (8-14d)**	**Late dead (15-21d)**	**Pipped**
		**----------------------------------(%)------------------------------**				
Control		4.73	4.71	0.80	4.43	1.62
SDP		5.65	3.13	0.60	2.87	1.24
SEM1		0.98	0.59	0.25	0.67	0.44
Age (weeks)						
29		6.62	3.55	0.96	2.20	1.67
45		4.52	3.11	0.20	3.30	0.99
63		4.44	5.09	0.94	5.46	1.64
SEM		1.20	0.73	0.31	0.82	0.54
	29	5.68	3.74	0.80	2.32^b^	1.94
Control	45	3.27	3.42	0.41	2.94^b^	0.87
	63	5.24	6.73	1.20	8.02^a^	2.06
	29	7.55	3.37	1.12	2.07^b^	1.39
SDP	45	5.76	2.80	1.62	3.66^b^	1.12
	63	3.63	3.21	0.67	2.89^b^	1.22
SEM		1.69	1.03	0.44	1.16	0.77
				Probability		
Breeder diet		0.51	0.06	0.56	0.10	0.54
Breeder Age		0.34	0.13	0.15	0.01	0.61
Breeder diet × age		0.43	0.19	0.58	0.02	0.76

^1^SEM: Standard error of the mean. a-b: Means within a column with different superscripts are different by the Tukey test (P < 0.05).

Post-hatch chick alterations (Table 4) were not influenced by the different treatments, except that chicks from 29-week-old hens had a higher percentage of red hocks (*P* < 0.05). As for chick weight, chicks from SDP supplemented hens had lower body weight at hatch (*P* < 0.05), and as the hen age increased, chick weight at hatch increased (*P* < 0.001). An interaction was found concerning chick length analysis, showing that chicks from SDP supplemented 45-week-old hens were slightly smaller than chicks from the control group of the same age hens (*P* < 0.001).

**Table 4:** Influence of feeding diets with spray-dried plasma (SDP) to broiler breeder hens on chick quality.

**Diet/age**		**Chick Weight (g)**	**Chick Length (cm)**	**Dehydrated**	**Navel problems**	**Red hocks**
Control		48.98^a^	18.06	0/300 (0%)	6/300 (2%)	4/300 (1.33%)
SDP		48.14^b^	17.99	1/300 (0.33%)	15/300 (5%)	6/300 (2%)
SEM1		0.19	0.03	0.001%	0.011%	0.007%
Age (weeks)						
29		43.01^c^	17.50^c^	1/200 (0.5%)	8/200 (4%)	8/200 (4%)^a^
45		50.78^b^	18.08^b^	0/200 (0%)	3/200 (1.5%)	2/200 (1%)^b^
63		51.89^a^	18.50^a^	0/200 (0%)	10/200 (5%)	0/200 (0%)^b^
SEM		0.23	0.04	0.003%	0.013%	0.009%
	29	43.28	17.43^d^	0/100 (0%)	2/100 (2%)	3/100 (3%)
Control	45	51.47	18.25^b^	0/100 (0%)	2/100 (2%)	1/100 (1%)
	63	52.19	18.5^a^	0/100 (0%)	2/100 (2%)	0/100 (0%)
	29	42.74	17.57^d^	1/100 (1%)	6/100 (6%)	5/100 (5%)
SDP	45	50.09	17.91^c^	0/100 (0%)	1/100 (1%)	1/100 (1%)
	63	51.60	18.5^a^	0/100 (0%)	8/100 (8%)	0/100 (0%)
SEM		0.33	0.05	0.003%	0.019%	0.013%
				Probability		
Breeder diet		0.001	0.13	0.99	0.13	0.99
Breeder Age		<.0001	<.0001	0.99	0.26	<.0001
Breeder diet × age		0.66	<.0001	0.99	0.29	0.99

^1^SEM: Standard error of the mean. a-c: Means within a column with different superscripts are different by the Tukey test (P < 0.05).

## Discussion

Given the rich concentration of proteins and active compounds in SDP, it was expected a substantial enhance in overall rooster semen quality, as these compounds could mitigate cell stress and maintain the intestinal integrity of roosters. In the present study, no effects regarding dietary SDP inclusion in rooster diets on semen volume, concentration, and motility characteristics were observed (Table 1). However, rooster age played an important role in sperm cell kinetics. The four progress parameters (VAP, VSL, STR, and LIN) and the two vigor parameters (VCL and BCF) were higher for the youngest rooster age group than for the older ages, while semen from 45-week-old roosters had the lowest values for the kinetic parameters among age groups. The diverse range of evaluated semen parameters such as the ones mentioned are associated with higher sperm motility (Farahi et al., 2018), which was also observed for 29-week-old roosters. The lowest sperm kinematics parameters from 45-week-old roosters were unexpected, as it is reported that rooster fertility decreases only at 72 weeks of age, after reaching a peak at 37 weeks (Cerolini et al., 1997; Weil et al., 1999). The reason for this finding could be the environmental temperature on the day of semen collection, as it was lower (10.7 ºC) than the comfort temperatures for roosters (Saeid and Al‐Soudi, 1975). Semen collected from roosters at 63 weeks of age having higher sperm concentration than semen collected from previous weeks was also unexpected. Previous studies indicate that as roosters age, lower sperm concentrations are found (Zhang et al., 1999; Haryuni et al., 2022), likely due to increased oxidative damage to testicles and reduced testosterone concentrations, thus lowering the quantity of sperm produced (Escorcia et al., 2020).

Semen from 45-week-old roosters showed fewer total defects than semen from 63-week-old roosters, regardless of SDP supplementation (*P* < 0.05). It has been reported that the total number of defects tends to be stable during 30 to 55 weeks of age, with increased values before and after this time period (Celeghini et al., 2001; Lagares et al., 2017), which was also observed in this study. However, SDP reduced the total number of sperm cell defects in the semen of 63-week-old roosters. Fertility reduction in aged roosters is often related to testicular atrophy (Lagares et al., 2017), abnormal morphology of efferent ducts (Kirby et al., 1990), and epididymal lithiasis (Janssen et al., 2000), with lithiasis causing chronic inflammation of the epididymal epithelium. Diets supplemented with SDP have been reported to increase serum IL-10 (Pérez-Bosque et al., 2016), the major anti-inflammatory cytokine, which may partially explain why SDP supplemented 63-week-old roosters produced fewer abnormal sperm cells than control birds. Although not measured in this study, dietary SDP could be modulating the immune system to reduce inflammation in the epididymal epithelium, leading to a decrease in reactive oxygen species release promoted by an inflammatory microenvironment that could potentially promote injury to the sperm cell. Past research has shown that dietary SDP can reduce pro-inflammatory cytokines and increase anti-inflammatory cytokine concentrations in the uterine mucosa of transport stressed pregnant mice, while also improving pregnancy rates (Song et al., 2015). Sperm cell defects over 20% can lead to a drastic fertility decrease (Surai and Wishart, 1996), and it is worth mentioning that semen from birds supplemented with dietary SDP produced sperm cells with a total sum of defects below 20%, which was not observed for the control group at 63 weeks.

The unsupplemented group also showed the highest value of middle piece abnormalities, while 45-week-old roosters and 63-week-old roosters supplemented with dietary SDP showed lower values, demonstrating potential for SDP supplementation to attenuate the bird age effect on sperm cell defects. SDP supplemented roosters also had lower sperm head and tail defects compared to the control group (*P* < 0.05). These results could also be explained by the high protein content and good amino acid profile of SDP (Granghelli et al., 2023), as amino acids and their metabolites are important nutritional factors for maintaining testicular evolution, as well as enhancing semen quality and fertility (Fouad et al., 2020).

Despite no differences between treatments being observed for infertility, early and middle embryonic deaths, and pipped egg rates, dietary inclusion of SDP for breeders was able to reduce the late embryonic death rate from eggs laid by 63-week-old birds. (Pérez-Bosque et al., 2016) states that SDP contains a diverse mixture of albumin, globulin, peptides, and growth factors, which are essential functional nutritional components with the potential to enhance hen production and embryo development in fertilized eggs. Some nutrients supplied in the breeder hen diet are deposited in the eggs and utilized for embryonic development, directly impacting chick quality at hatch and subsequent post-hatch performance (Hasselquist and Nilsson, 2009; Araujo et al., 2018).

The rapid development of bird embryos in a short time (Deeming and Pike, 2013) can lead to high physiological stress, which, if not adequately managed, can have detrimental effects on embryo growth and survival. Under physiological stress, commercial bird embryos are prone to high oxidative damage during development if there is no effective nutritional strategy to counteract it. Consequently, it is possible that SDP reduced the negative consequences of stress, resulting in decreased late-stage embryonic mortality.

No changes regarding chick dehydration, navel problems, or red hocks were observed between dietary treatments, although chicks from 29-week-old hens showed a higher incidence of red hocks. This contradicts the findings of Ulmer-Franco et al (Ulmer-Franco et al., 2010), who reported fewer culled chicks (due to unhealed navels, red hocks, weakness, or physical abnormalities) from 29-week hens. Eggs from older hens contain more yolk available for the embryo (Suarez et al., 1997), explaining the increase in chick weight at hatch as hen age increases. However, it is also reported that albumen quality in eggs decreases with increased breeder age, which may regress chick quality (Narinç and Aydemir, 2021). It is important to note that hock quality does not always result in worse performance during the broiler productive cycle (Silva et al., 2021).

In the present study, unsupplemented breeders had chicks with increased length as breeder age progressed. However, 45-week-old SDP supplemented breeders had offspring with shorter lengths than control breeders of the same age. Despite this finding, previous studies show a weak correlation between chick length and chick weight (Willemsen et al., 2008). Additionally, egg weight loss and at-hatch dehydration could explain the observed results (Deeming, 2005).

## Conclusion

In conclusion, it is conceivable to assert that 1% SDP in feed for breeder hens and roosters can enhance the reproductive performance of roosters by decreasing the number of sperm cell defects, mainly in aged birds, as well as decreasing late embryonic mortality in aged hens.

## Supplementary Material

Supplementary material accompanies this paper.

Caption S1 Rooster semen settings to be used within CASA software.

This material is available as part of the online article from https://doi.org/10.1590/1984-3143-AR2024-0111
